# Supplementary material for: Attachment beyond the screen: The influences of demographic factors and parasocial relationships on social media use in Qatar
Source: PLoS One. 2025 Jun 20;20(6):e0326685. doi: 10.1371/journal.pone.0326685 (PMC12180621; doi:10.1371/journal.pone.0326685)
Supplement: Attachment — Submitted filename: 20241206 Reviewer 2 plos one.docx [file pone.0326685.s002.docx]

**Letter of responses to Reviewer 2**

Dear Reviewer,

Thank you very much for spending a great amount of time and effort reviewing our manuscript. Your detailed comments have helped us improve the quality of our paper.

In the revised manuscript, the revised parts are highlighted in yellow. In this letter, *your comments are in italic*, and **our answers are in bold**.

*****

**Reviewer 2**

*In section 1.1.Excessive social media use and its outcomes:It would be better to include statistics on Non-WEIRD countries if it is possible.*

**Thank you for your suggestion! We have added additional information as well as research findings about social media use in Non-WEIRD countries. Please see below:**

[…]
However, findings from WEIRD contexts may not directly generalize to non-WEIRD populations, because various cultural, socioeconomic, and social norms might significantly influence the social media use pattern, and the formation and impact of parasocial relationships. Currently, studies on social media use in non-WERID contexts offered complicated findings. For example, Chinese netizens average about 2 hours per day social media use time [26]. One study on Chinese adolescents suggested that social media use time alone is not associated with increased anxiety toward their body image. However, when seeking attention through the use of social media, the more social media use time Chinese adolescents, the greater they would suffer from social appearance anxiety [27]. On the other hand, India has more than 600 million social media users [28], one studies on Indian adolescents offered similar findings, suggesting that long time use of social media is associated with increased stress, anxiety, and depression [29]. However, few studies have been conducted on non-WEIRD populations in the Middle East. One cross-cultural study examined the mindful use of social media among Iranian and American users, finding that Iranian participants reported higher levels of mindful social media use, which were associated with lower symptoms of social media addiction [30]. Furthermore, one recent study investigated the role of parasocial relationships with favorite food influencers among Iranian social media users. Their study found that stronger PSRs with food influencers were associated with higher levels of eating disorder symptoms, food addiction, and grazing behaviors [31]. However, the aforementioned studies primarily examined social media use and parasocial relationships (PSRs) within Iran, leaving a significant gap in research on non-WEIRD populations in the broader Middle Eastern context. Qatar’s unique context offers a distinctive environment where rapid economic development and high internet penetration coexist with deeply rooted cultural and traditional norms. This blend creates a unique dynamic in how individuals engage with social media and form PSRs with influencers.

[…]

*2. Is there any research that has conducted on your research variables in Non-WEIRD countries that can support your findings? If there is, you can use it in the discussion section.*

**Thank you for your input. We have added additional references to connect the current study findings with prior studies. Please see below**

[…]

The finding that a higher PSR degree with Instagram influencer(s) is associated with higher daily social media usage time is in alignment with prior studies [22, 23].

[…]

The present study suggests that cultural and societal norms can have a considerable background influence in shaping the dynamics of social media usage behavior, which is in alignment with extant studies [27, 62, 63].

[…]

*3. The authors of this article are expected to explain that why the data collection took place in 2020 and the article was written about 4 years later.*

**Thank you for the question. The original dataset was collected by a separate research team which is not associated with us. The dataset was deposited openly as a peer-reviewed data article; however, we found critical gaps in the former analyses/interpretations of that original data while conducting preliminary background research for our social media study project on Asian contexts. Assessing that the collection period of the dataset was sufficiently recent and relevant regarding the modern online landscape, we decided to conduct our analysis on this dataset using a different approach as well as method, targeting the aforementioned gaps in understanding. This was done in the spirit of data transparency and the advocacy for scientific integrity and reproducibility.**

*4. It is suggested that additional editing be done on the paper to meet the criteria of standard English language.*

**Thank you for your suggestion! We have gone through another round of language check.**
