# Supplementary material for: Attachment beyond the screen: The influences of demographic factors and parasocial relationships on social media use in Qatar
Source: PLoS One. 2025 Jun 20;20(6):e0326685. doi: 10.1371/journal.pone.0326685 (PMC12180621; doi:10.1371/journal.pone.0326685)
Supplement: Attachment — Submitted filename: 20250422 reviewer 3 plos one.docx [file pone.0326685.s003.docx]

**Letter of responses to Reviewer 3**

Dear Reviewer,

Thank you very much for spending a great amount of time and effort reviewing our manuscript. Your detailed comments have helped us improve the quality of our paper.

In the revised manuscript, the revised parts are highlighted in yellow. In this letter, *your comments are in italic*, and **our answers are in bold**.

*****

**Reviewer 3**

*Thank you for the opportunity to review the revised manuscript. This study presents a valuable contribution to the literature on social media behavior by focusing on a non-WEIRD population in Qatar; an underexplored yet important context. The manuscript is well-written, and the authors have clearly made substantial revisions in response to earlier feedback.*

**Thank you for your kind words.**

*That said, several areas still require clarification or refinement. Here is my detailed feedback:
1. The manuscript mentions use of a six-item PSR scale but does not present the items or explain how they were adapted for this cultural context. Given the centrality of this construct, please consider including the items in an appendix or table.*

**Thank you for your academic rigor. We have added the Questionnaire at the end of the manuscript, highlighting the items used in this study to measure PSR. Please see below:**

**Questionnaire in English**

**Part A: General Information**

**1. How many hours do you spend on social media every day?**

🞏Never	🞏 1-2 hours	🞏 3-4 hours	🞏 5 hours or above

**2. Which Instagram influencer do you mostly follow? (mention one influencer)**

🞏 --------------------------------

**3. When did you start following this Instagram influencer?**

🞏 less than 6 months ago

🞏 One year ago

🞏 Two years ago

🞏 Three years ago or more

**4. What is the area of expertise of this Instagram influencer?**

🞏 Fashion

🞏 Traveling

🞏 Beauty products

🞏 Food and beverages

🞏 Others: --------------------------------

**Part B: Rating Statements**

**To what extent do you agree on the following statements?**

(1) Strongly disagree, (2) Disagree (3) Neutral (4) Agree (5) Strongly agree

| Statement | 1 | 2 | 3 | 4 | 5 |
| --- | --- | --- | --- | --- | --- |
| Hom1: This Instagram influencer thinks like me. |  |  |  |  |  |
| Hom2: This Instagram influencer is similar to me. |  |  |  |  |  |
| Hom3: This Instagram influencer is like me. |  |  |  |  |  |
| Hom4: This Instagram influencer shares my values. |  |  |  |  |  |
| Hom5: This Instagram influencer has a lot in common with me. |  |  |  |  |  |
| Hom6: Instagram influencer behaves like me. |  |  |  |  |  |
| Hom7: This Instagram influencer has thoughts and ideas that are similar to mine. |  |  |  |  |  |
| Hom8: I think that my Instagram influencer could be a friend of mine. |  |  |  |  |  |
| Hom9: I would like to have a friendly chat with my Instagram influencer. |  |  |  |  |  |
| Pop1: This Instagram influencer has a high exposure in the Instagram environment. |  |  |  |  |  |
| Pop2: This Instagram influencer has a high popularity in the Instagram environment. |  |  |  |  |  |
| Pop3: This Instagram influencer has a high reputation in the Instagram environment. |  |  |  |  |  |
| Lev1: This Instagram influencer can cause debate in the Instagram environment. |  |  |  |  |  |
| Lev2: This Instagram influencer is topical in the Instagram environment. |  |  |  |  |  |
| Lev3: This Instagram influencer remarks in the Instagram environment are sensational. |  |  |  |  |  |
| Fash1: This Instagram influencer can lead the trend in the Instagram environment. |  |  |  |  |  |
| Fash2: This Instagram influencer is very fashionable. |  |  |  |  |  |
| Fash3: This Instagram influencer is very sensitive to fashion. |  |  |  |  |  |
| Aff1: This Instagram influencer is very close to people. |  |  |  |  |  |
| Aff2: This Instagram influencer behaviour is in a popular style. |  |  |  |  |  |
| Aff3: This Instagram influencer is a very down-to-earth person. |  |  |  |  |  |
| PSI1: I feel close enough to my favourite Instagram influencer to use his(her) Instagram. |  |  |  |  |  |
| PSI2: I feel comfortable about my favourite Instagram influencer messages. |  |  |  |  |  |
| PSI3: I can rely on information I get from my favourite Instagram influencer. |  |  |  |  |  |
| PSI4: I feel fascinated with my favourite Instagram influencer’s Instagram. |  |  |  |  |  |
| PSI5: In the past, I pitied my favourite Instagram influencer when he/she made a mistake on his/her Instagram. |  |  |  |  |  |
| PSI6: I think that my favourite Instagram influencer’s Instagram is helpful for my interests (in fashion and others). |  |  |  |  |  |
| WOM1: I am likely to say positive things about what my Instagram influencer promotes to others. |  |  |  |  |  |
| WOM2: I would recommend what my Instagram influencer promotes to my friends and relatives. |  |  |  |  |  |
| WOM3: If my friends were looking for a product or service of this type, would recommend what my Instagram influencer said about it. |  |  |  |  |  |
| Int1: I will buy the product or the service that Instagram influencer promoted through Instagram. |  |  |  |  |  |
| Int2: I have the intention to buy the product or the service that my Instagram influencer promoted on Instagram. |  |  |  |  |  |
| Int3: I am interested in buying the product or the service my Instagram influencer promoted on Instagram. |  |  |  |  |  |
| Int4: It is likely that I will buy the products or services my Instagram influencer promoted on Instagram in the future. |  |  |  |  |  |
| Int5: Overall, I am pleased with what my Instagram influencer promotes on Instagram. |  |  |  |  |  |

**Part C: Demographic Information**

**1. What is your age?**

🞏18 - 24 years old	🞏25 - 34 years old	🞏 35 - 44 years old

🞏 45 - 54 years old	🞏55 - 64 years old	🞏 64 years & above

**2. What is your gender?**

🞏 Male	🞏 Female

**3. What is your nationality?**

🞏Qatari	🞏Non-Qatari

**6. What is your annual income?**

🞏	Less than 50,000 Qatari Riyals

🞏	50,001-150,000 Qatari Riyals

🞏	150,001-250,000 Qatari Riyals

🞏	250,001-350,000 Qatari Riyals

🞏	350,001-450,000 Qatari Riyals

🞏	450,001 Qatari Riyals or above

*2. At various points (e.g., “PSRs cause increased social media use”), causal implications are made. Please rephrase to emphasize correlational interpretation throughout. This is particularly important given the cross-sectional nature of the data.*

**Yes, you are right, and we are thankful for your insightful comments. We have adjusted our wording to address your concerns.**

…

**Conclusions:** This study highlights that demographic factors such as gender, age, and income in their interactions with parasocial relationships are associated with social media usage time within the non-WEIRD social context of Qatar. The findings underscore the necessity of considering the specific local cultural settings when studying social media behaviors.

…

In the case of social media users, susceptible demographic groups may desire to gratify various social identity-based needs through interactions with influencers and celebrities, which might be associated with extensive social media usage time and PSRs.

*…*

This personalized content loop would be associated with increased social media usage, as users are drawn into a continuous cycle of engagement [27]. Reversely, as users spend more time on social media, there is a higher chance for them to come across influencers’ content that is engaging and stimulating, which helps form or reinforce the perceived connections between followers and influencers [49]. The felt attachment due to various psychological factors/reasons, as suggested in the UGT [24, 25], can further strengthen a PSR through this reciprocal loop of social media content engagement.

*…*

Regarding PSRs among younger social media users, the formation of tightly-knit fandom communities or subcultures, where members often use internet slang and unique communication patterns extensively [60] can increase the appeal toward in-group engagement. This may reinforce the sense of belongingness and commitment, and thus being associated with greater usage time.

… *3. The dataset was collected in early 2020. Although the authors now mention this and explain their rationale for use, I recommend briefly reflecting on how Instagram use or influencer engagement in Qatar may have evolved since that time—particularly given the impact of COVID-19 on digital behavior.*

**Thank you for your suggestion! We have added additional information regarding Instagram development in Qatar. Please see below:**

*…*

In fact, recent estimates suggest that Instagram’s penetration in Qatar grew by over 10% between 2020 and 2022, and influencers took on a more prominent role in disseminating public information and shaping consumer habits during the pandemic [4,5]. As of January 2024, Instagram users have been reported to reach 1.7 million in 2024 (up from 1.1 million in 2023) with 35.1% female users and 64.9% male users[33].

As a result of the limited research on non‑WEIRD Middle Eastern populations and the rapid, COVID‑19–induced shifts in Instagram adoption and influencer engagement, understanding these dynamics in Qatar’s digital spaces not only contributes to the social media development in Qatar but also offers novel insights into similar regions of quickly developing or emerging economies, where technological development and modernization meet with conservative values and ideologies.

… *4. The Discussion is well developed, however, some parts of it just repeat earlier findings without sufficient interpretation. I suggest revising the Discussion further to focus more on implications and conceptual takeaways rather than just repeating the results.
5. In the future research directions, some further limitations should be addressed. For instance, the inclusion of influencer characteristics (e.g., gender, genre, celebrity status) could be explored in future PSR studies. Also, the potential for longitudinal or qualitative research to deepen understanding of user-influencer dynamics should be highlighted here.*

**We thank you for your thoughtful feedback. Now we have made revisions in the implications and future studies section:**

**Implications**

The present study suggests that cultural and societal norms can have a considerable background influence in shaping the dynamics of social media usage behavior, which is in alignment with extant studies [27, 65, 66]. From a practical standpoint, the results suggest that interventions aimed at reducing excessive social media use should be tailored to the specific demographic and cultural context of the target population. In the case of Qatar, addressing the unique local characteristics of women, young generations, or lower-income groups might help increase effectiveness.

To be more specific, given that females with strong PSRs tend to use social media more extensively, policy makers could feature relatable female role models or influencers to promote balanced online–offline lifestyles. Such campaigns can speak directly to women’s experiences, showing positive ways of engaging with influencers (e.g., seeking inspiration without excessive scrolling) while acknowledging underlying social pressures.

Also, because the findings suggest that younger users are especially prone to both higher PSRs and higher social media usage, programs that teach digital literacy and self-regulation (e.g., how to set healthy screen-time boundaries) could be integrated into high school or university curricula. These initiatives can reduce the risks of problematic use by encouraging purposeful engagement that still supports healthy identity exploration and peer bonding.

Lastly, for lower-income individuals who may rely heavily on cost-free entertainment such as social media, policymakers and local organizations could facilitate affordable offline social activities or community events. For example, subsidized access to sports clubs, public libraries, or cultural festivals can offer enjoyable and meaningful non-digital outlets.

Future work could explore how influencer-specific characteristics—such as gender, genre, or celebrity status—shape parasocial relationships (PSRs) and social media usage. Longitudinal and qualitative approaches may further deepen our understanding of how user–influencer dynamics evolve over time, especially in non-WEIRD contexts where distinct cultural factors play a key role. *6. Line 116: “usa time” should be corrected to “usage time.”*

**We are sorry for this typo and the issue has been fixed.**

*7. Consider adding a brief explanation of why MCMC was preferred over frequentist approaches for readers unfamiliar with Bayesian methods..*

**Yes, this is indeed thoughtful to enhance the manuscript’s readability. We have added additional explanations why MCMC was preferred.**

**…**

the high skewness in demographic factors (due to the nature of digital social media use) can negatively affect inference accuracy because of the low data points available in some categories. For example, because of the higher proportion of women (61.7% vs. 38.3% men), traditional frequentist analyses can yield less stable parameter estimates under such imbalances or low data counts in specific subgroups. By contrast, a Bayesian framework aided by MCMC simulations allows for more flexible handling of skewed data, as it models parameters as probability distributions rather than fixed values. This approach “borrows strength” from more populated subgroups while explicitly accounting for uncertainty in underrepresented categories.

**We sincerely thank the reviewer for the thoughtful comments and valuable suggestions, which have substantially improved the clarity and depth of this manuscript. The insights prompted us to expand our discussion on the implications of cultural and societal norms, refine our methodological justifications, and outline further avenues for investigation. We appreciate the time and effort invested in reviewing our work, and we look forward to continuing this scholarly dialogue.**
